# Lap. Nissen fundoplication leads to better respiratory symptom control than Toupet in the long term of 20 years

**DOI:** 10.1007/s00423-023-03108-8

**Published:** 2023-09-22

**Authors:** Philipp Gehwolf, Teresa Agerer, Nadine Stacul, Katrin Kienzl-Wagner, Aline Schäfer, Valeria Berchtold, Fergül Cakar-Beck, Gasser Elisabeth, Heinz Wykypiel

**Affiliations:** Department of Visceral, Transplant, and Thoracic Surgery (VTT), Center of Operative Medicine, Medical University of Innsbruck (MUI), 6020 Innsbruck, Austria

**Keywords:** Humans, Fundoplication, Heartburn, Deglutition disorders, Quality of life, Laparoscopy, Gastroesophageal reflux

## Abstract

**Introduction:**

Having performed anti-reflux surgery for thirty years, it was important to reexamine our patients in the long term to enlarge the body of evidence concerning classical and extraesophageal symptoms that are differently controlled by Nissen or Toupet fundoplication.

**Objectives:**

We report a cohort of 155 GERD patients who underwent fundoplication within a tailored approach between 1994 and 2000. Changes in the perioperative functional outcome, GERD symptoms, and quality of life are being analyzed 10 and 20 years after the operation.

**Results:**

The operation resulted in a superior quality of life compared to a patient cohort treated with PPI therapy. We found that both surgical methods (laparoscopic Nissen fundoplication and laparoscopic Toupet fundoplication) cure classical symptoms equally (heartburn, regurgitation, and dysphagia). GERD patients receiving a Toupet fundoplication seem more likely to suffer from extraesophageal GERD symptoms 10 and 20 years after surgery than patients with a Nissen fundoplication. On the other hand, some patients with Nissen fundoplication report dysphagia even 10 and 20 years after surgery.

**Conclusion:**

Both the laparoscopic Nissen and Toupet fundoplications provide excellent symptom control in the long term. Moreover, the Nissen fundoplication seems to be superior in controlling extraesophageal reflux symptoms, but at the expense of dysphagia. In summary, tailoring the operation based on symptoms seems advantageous.

## Introduction

Surgical procedures are known to successfully cure GERD in about 85–93% of patients in short- and midterm observation periods [1]. However, dysphagia can occur postoperatively, especially after a Nissen fundoplication. In the mid-1970s, this experience transformed the classical Nissen fundoplication into a “floppy Nissen” by Donahue et al. [2]. With the onset of the laparoscopic era in the early 1990s, surgical methods were adapted and modified again [3]. An alternative to laparoscopic Nissen fundoplication was seen in Toupet’s laparoscopic partial posterior fundoplication to overcome postoperative dysphagia. Therefore, the indication for a Toupet fundoplication in GERD patients was based on the grade of motility impairment on esophageal manometry [3]. Through this “tailoring,” patients with limited motility were allocated to a laparoscopic Toupet fundoplication and those with normal motility to a Nissen fundoplication [4]. Long-term follow-up studies confirmed a lower rate of dysphagia and other side effects after Toupet Fundoplication with similarly good symptom control [5, 6]. As a result, Toupet fundoplication was considered the “one fits-all” solution for all patients, regardless of motility.

After a Toupet fundoplication in all patients, the question arises whether the control of every particular esophageal and extraesophageal GERD symptom is sufficient. There are hardly any long-term studies that investigate this question, but some recent publications show that control of extraesophageal symptoms after a Nissen may be superior to the Toupet operation [7, 8].

Since we have been performing anti-reflux surgery (ARS) since the 1990s, it was worth reexamining our early patients with a focus on classical and extraesophageal symptom control in the long term.

## Objectives

We report a cohort of 155 GERD patients who underwent fundoplication within a tailored approach between 1994 and 2000 in a tertiary referral center. Changes in the perioperative functional outcome, GERD symptoms, and quality of life were analyzed 10 and 20 years after the operation. Additionally, esophageal and extraesophageal symptom control in laparoscopic Nissen fundoplication (LNF) and laparoscopic Toupet fundoplication (LTF) were compared at 10 and 20 years after surgery.

## Patients and methods

**Fig. 1:**
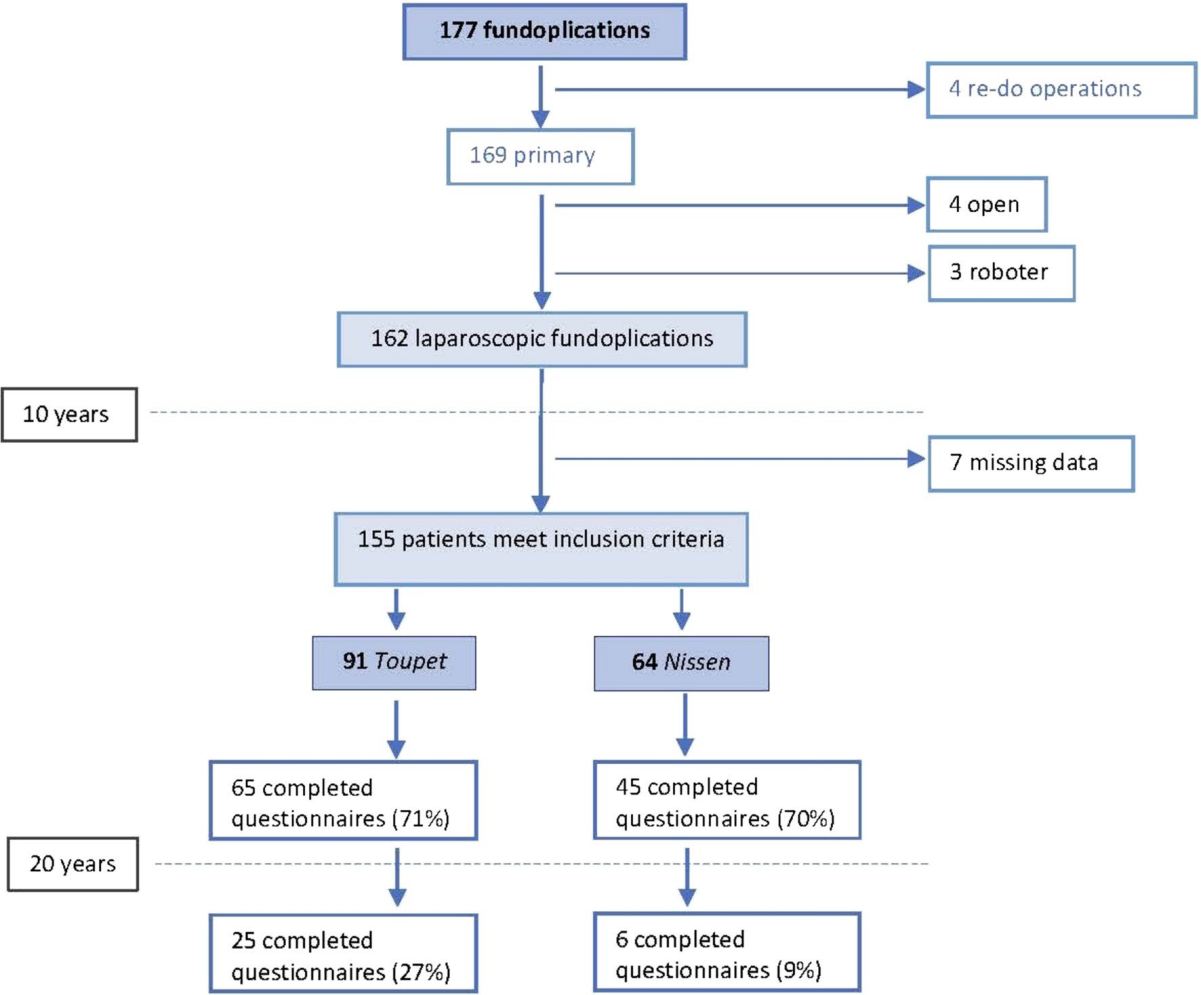
Flow diagram (exclusion criteria) of surgical GERD patients and return rate of questionnaires over a period of 20 years

A total of 177 GERD patients, operated at the Medical University of Innsbruck between January 1994 and December 2000, were included in this retrospective single-center analysis. Exclusion criteria were refundoplication, open fundoplication, robotic assisted operation, missing consent to answer the questions, and no complete questionnaire at ten-year follow-up (Fig. 1).

All patients had a preoperative workup including endoscopy with biopsies, barium swallow, and esophageal manometry (water-perfused stationary pull-back technique, Mui Scientific, Ontario, Canada), as well as esophageal 24-h impedance pH monitoring (Sleuth® multi-impedance pH monitoring system, Sandhill Scientific, Highlands Ranch, CO, United States). For grading erosive esophagitis, the Savary–Miller system was used [9]. Barrett’s metaplasia was defined as the presence of presence of alcain-positive goblet cells in histology in the columnar epithelium of the distal esophagus. In our reporting system, Barrett’s was not included in Savary–Miller grade IV but was reported separately. Hiatal hernias were graded as Type I–IV according to the Siewert classification [10].

GERD symptoms were recorded in detail with a symptom questionnaire (Appendix 1). Patients with impaired motility on manometry (more than 20% defective peristalsis) underwent a Toupet fundoplication, whereas patients with physiologic esophageal motility obtained a Nissen fundoplication, the so-called tailored approach [11]. The lower esophageal sphincter (LES) was evaluated in manometry with a water-perfused catheter (Mui Scientific, Ontario, Canada) using the stepwise pullback technique [12].The LES was defined as incompetent if the mean resting pressure at the respiratory inversion point was below 8 mmHg and/or if the intraabdominal sphincter length was below 1.2 cm. Esophageal peristalsis was defined as defective if more than 20% of the esophageal contractions of ten swallows of 5 ml of tap water were below 30 mmHg (ineffective clearance function), or if the contractions occurred simultaneously (propagation > 20 mm/s), or if the propagation was interrupted (contraction amplitude < 15 mmHg) [13]. The surgical technique for laparoscopic Nissen and Toupet fundoplication was described previously [11, 13–15]. In short, generous dissection up into the mediastinum in order to create a tension-free intra-abdominal esophagus length of 3 cm, closure of the crura with single nonabsorbable sutures, calibrated with a 36F gastric tube in place. The creation of a 2-cm fundic wrap in the Nissen fundoplication was accomplished using a six-point U-shaped stitch buttressed by Teflon patches. This U-stitch always incorporated the esophagus. Sutures not passing through the esophageal wall were placed above and below the U-stitch. For the creation of the partial posterior fundoplication, the right limb of the fundic wrap was fixed to the right crus using three stitches, and then, its edge was sutured to the right anterior aspect of the esophagus using another three sutures. The left limb of the fundic wrap was fixed to the left anterior aspect of the esophagus using three sutures over a length of 3 cm. Approximately 1 cm of the anterior wall of the esophagus was left unwrapped, resulting in a 270° wrap, which is different from the originally reported 180° Toupet wrap. In both procedures, nonabsorbable sutures were used (Ethibond 2–0, Ethicon/Johnson & Johnson, Hamburg, Germany). The posterior trunk of the vagus nerve was always left within the fundic wrap.

Six months after the operation, endoscopy, esophageal manometry, and 24-h impedance pH monitoring were repeated.

### Quality of life assessment

For quality-of-life assessment, a standardized and validated general health questionnaire (medical outcomes study short form (SF-36)) was used. It yields an eight-scale profile of scores as well as physical and mental health summary measures and is a generic measure as opposed to one that targets a specific age, disease, or treatment group [16, 17]. Moreover, a standardized and validated gastrointestinal quality of life questionnaire (Gastrointestinal Quality of Life Index, GILQI) by Eypasch et al. [18] was handed out in order to take a closer look at effect size and relative efficiency [19].

### Symptoms and patients’ personal appraisal

A self-designed detailed symptom questionnaire with the possibility of a personal statement by the patient was handed out preoperatively and 10 and 20 years after surgery. This questionnaire is not standardized and combines qualitative and quantitative data collection through patient self-reporting. Several symptoms were asked about the frequency of their occurrence, with the possible answers: never, occasionally (once or several times per month), and often (once or several times per week, and daily). There are also questions about impairments in everyday life, general satisfaction, and the possibility of writing down unrecorded symptoms (Appendix 1). Dysphagia was scored according to the following grading system: mild (easily tolerated, slower eating without weight loss), moderate (interfering with lifestyle, no weight loss), and severe (with weight loss) [20]. Self-reported mild dysphagia and no dysphagia were summarized as no dysphagia, whereas moderate (1–2 times per week) and severe dysphagia (daily) were summarized as existing dysphagia.

### Data and statistics

Statistical analysis of the data collected in this study was performed using Microsoft® Excel V. 16 and GraphPad Software® Prism V9. For categorical variables, the chi-square test was used; for non-normally distributed metric or ordinal scaling variables, the paired Wilcoxon test was used. For non-compared data, descriptive statistics were used. Dependent on appropriateness, numbers are depicted in median with percentiles (5–95%), or mean and standard deviation, respectively.

## Results

### Patient characteristics

A total of 155 GERD patients out of 177 meet the inclusion criteria. Manometry showed that 91 patients had poor esophageal motility, so a laparoscopic Toupet fundoplication (LTF) was done. A laparoscopic Nissen fundoplication (LNF) was performed on 64 patients, who had normal esophageal motility. In the LTF group, 33 patients were female (36.3%); in the LNF group, 22 (34.4%) were female; all in all, we had 55 female patients (35.5%). At the time of operation, patients were median 49 years old (28–71). They had a history of GERD for a median of 10 years (1.5–30). Erosive esophagitis was found in 149 (96%) patients.

Barrett’s metaplasia was found in 93 (60%) patients, independent from the esophagitis stage. In seven patients (4.5%), no endoscopic sign of esophagitis was reported. A hiatal hernia [10] was present in 152 (98%) patients. Three patients (1.9%) had GERD without a hiatal hernia. A detailed report is displayed in Table 1.

**Table 1:** Patients’ characteristics. f: female; m: male; LTF: laparoscopic Toupet fundoplication; LNF: laparoscopic Nissen fundoplication. Erosive esophagitis is defined according to the Savary–Miller staging system [9], Barrett’s is defined as intestinal columnar cell metaplasia with the presence of goblet cells on histology, hiatal hernia is classified in 4 types [36]: I sliding hiatal hernia and II–IV paraesophageal hernia, whereas in type II the stomach migrates into the chest and ‘‘rolls’’ over the esophagus with the gastroesophageal junction still laying down into the abdomen; type III occurs when the stomach migrates into the chest and ‘‘rolls’’ over the esophagus with the concomitant migration of the gastroesophageal junction into the chest; type IV occurs when, together with the stomach, there is herniation of other intra-abdominal contents through the hiatus [37]

patient's characteristics					
		median	(5%-95%)		
Age (years)		49	(28–71)		
Duration of symptoms (years)		10	(1,5–30)		
Sex		*n*			
	f	55	(LTF: 33; 36, 3%)	(LNF 22; 34, 4%)	
	m	100			
Erosive Esophagitis		pre-OP (*n*)	%	post-OP (*n*)	%
	undef.	51	32, 9	36	23, 2
	0	7	4, 5	117	75, 5
	I	11	7, 1	1	0, 6
	II	37	23, 9	0	0, 0
	III	10	6, 5	0	0, 0
	IV	39	25, 2	1	0, 6
	(Barrett’s)	93		80	
Hiatal hernia	0	3	1, 9		
	I	124	80, 0		
	II	0	0, 0		
	III	26	16, 8		
	IV	2	1, 3		
Laparoscopic Toupet Fundoplication				91	
Laparoscopic Nissen Fundoplication				64	

Out of the 155 operated patients, 110 (71%) completed the questionnaires ten years after the operation and 31 (20%) twenty years after surgery.

### Mortality and morbidity

Perioperative (90-day) mortality corresponded to zero. 145 patients (93.5%) did not have any complications. Minor complications (Clavien–Dindo < IIIa) occurred in 6 patients (3.9%): two thromboses, two cases of pneumonia, one not specified respiratory complication, and one persistent pneumothorax. Major complications (Clavien–Dindo > IIIa) occurred in four patients: three reoperations because of bleeding, empyema, a gastric tear, and one respiratory insufficiency, with the need for reintubation and treatment at the intensive care unit. Five patients needed a reoperation due to hernia recurrence within the first 10 years of follow-up. It is important to note that none of these patients developed esophageal cancer.

### Anti-reflux barrier

**Fig. 2:**
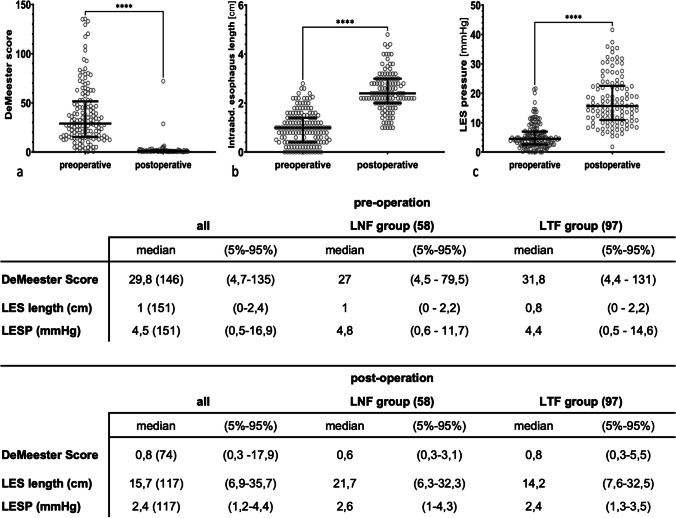
Characteristics of the anti-reflux barrier displayed as a plot and table. **a** Significant difference (*p* 0.0001) between the DeMeester Score before and after surgery. **b** Significant difference (*p* 0.0001) between the intraabdominal esophagus length (in cm) before and after surgery. **c** Significant difference (*p* 0.0001) between the lower esophageal sphincter (LES) pressure (in mmHg) before and after surgery. **d** Differentiated data for LNF and LTF in median and percentile (5–95%)

Preoperatively, the LESP was in the median range of 4.5 mmHg (0.5–1.9), the intraabdominal esophageal length was in the median range of 1 cm (0–2.4), and the DeMeester score was in median 29.8 (4.7–135). In all patients, the anti-reflux barrier could be reestablished. Six months after surgery, in 117 patients, the LESP was in median 15.7 mmHg (6.9–35.7; *p* 0.001), the intra-abdominal esophageal length in median 2.4 cm (1.2–4.4; p 0.001) and in 74 patients the DeMeester score was in median 0.8 (2.4–14.2; p 0,001) (Fig. 2).

### Symptoms

*Preoperatively*, most of GERD patients suffered from classic esophageal symptoms. Nearly all patients reported heartburn; most of them regurgitation, and about a quarter also dysphagia. The main extraesophageal symptoms were cough, chronic throat clearing, and globus sensation. Hoarseness and unspecific non-cardiac chest pain were rather rare symptoms (Table 2).

**Table 2:** GERD symptoms pre- and post-operation *LTF* laparoscopic Toupet fundoplication, *LNF* laparoscopic Nissen fundoplication

	LTF						LNF					
	pre-op		10a post-op		20a post-op		pre-op		10a post-op		20a post-op	
Esophageal symptoms												
Heartburn	62	95%	10	15%	4*	16%*	42	93%	4	9%	1**	17%**
Dysphagia	15	23%	3	5%	8*	32%*	11	24%	11	24%	1**	17%**
Regurgitation	49	75%	7	11%	8*	32%*	38	84%	5	11%	2**	33%**
Extraesophageal symptoms												
Globus sensation	7	11%	10	15%	8*	32%*	7	16%	4	4%	0**	0%**
Cough/ chronic throat clearing	14	22%	14	22%	8*	32%*	8	18%	1	2%	2**	33%**
Hoarseness	5	8%	9	14%	10*	40%*	5	11%	3	7%	3**	50%**
Non cardiac chest pain	4	6%	9	14%	9*	36%*	3	7%	3	7%	1**	17%**

^*^n = 25

^**^n = 6

#### Classic esophageal GERD symptoms postoperatively

Heartburn and regurgitation were well controlled after surgery in both groups. In the LTF group 10 years after surgery, 15% of GERD patients reported heartburn, compared to 9% in the LNF group. Twenty years after the operation, 16% and 17% reported heartburn, respectively. Regurgitation was reported by 7 patients in the LTF group and 5 in the LNF group 10 years after surgery but reoccurred in 32% and 33% twenty years after operation, respectively. Dysphagia was initially well controlled in the LTF group (5%). In contrast, 24% of the patients in the LNF group reported dysphagia ten years after operation. However, the rates increase twenty years after operation (32% LTF and 17% LNF (Table 2).

#### Extraesophageal GERD symptoms postoperatively

Cough (22%), globus sensation (15%), hoarseness (14%), and unspecific non-cardiac chest pain (14%) were reported in the LTF group ten years after operation, rising up to cough (32%), globus sensation (32%), hoarseness (40%), and unspecific non-cardiac chest pain (36%) twenty years after operation. Cough, globus sensation, hoarseness, and chest pain occurred in 2–7% in the LNF group ten years after operation and in 17–50% twenty years after surgery (Table 2).

#### Quality of life

**Fig. 3:**
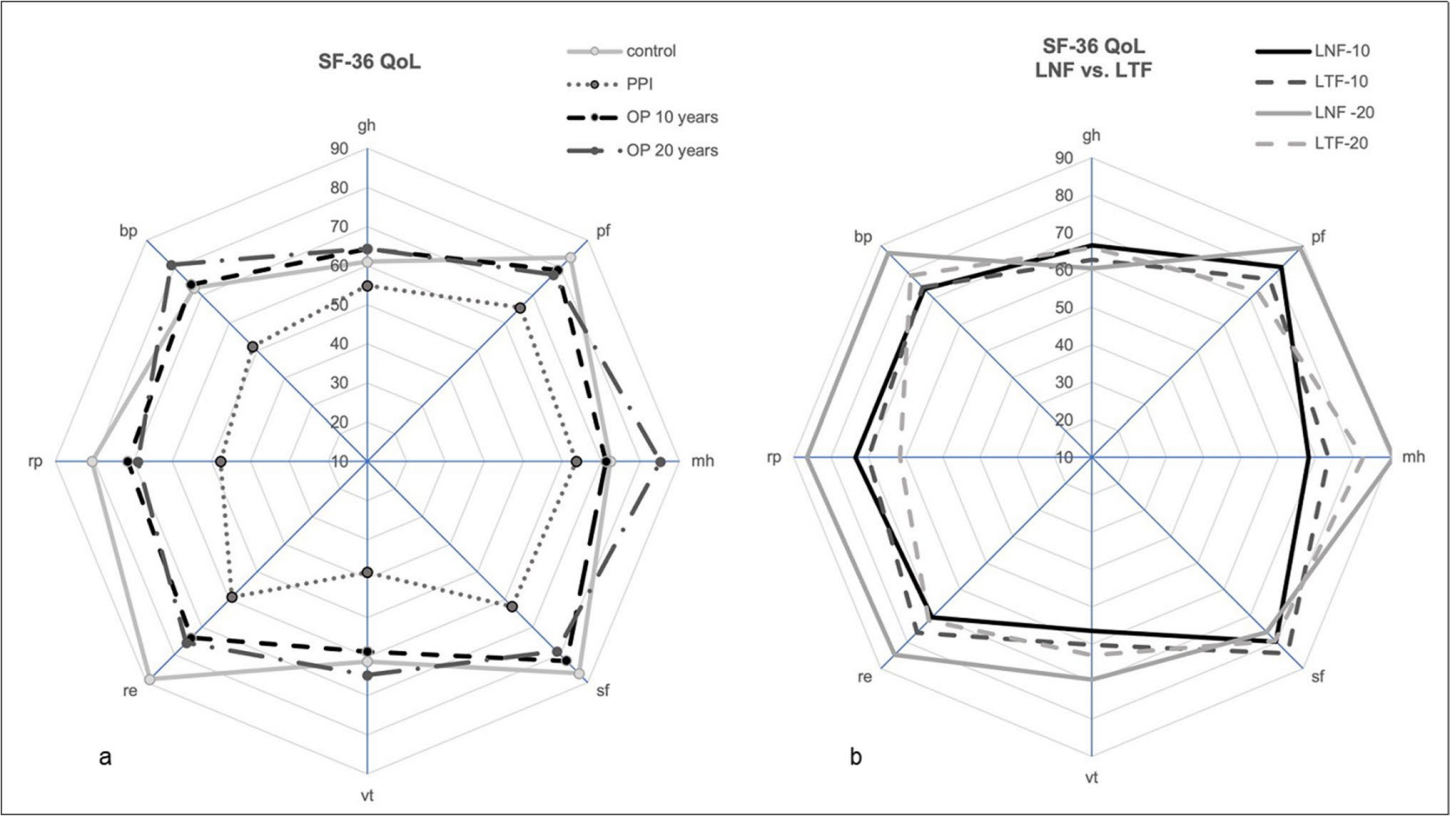
**a** Quality of life according to the 36-item short-form health survey questionnaire 10 and 20 years after operation in comparison to a healthy control population (control) and a standardized medically treated group (PPI) [20, 21]. Each item is evaluated on a 0–100 scale. gh: general health; pf: physical functioning; mh: mental health; sf: social functioning; vt: vitality; re: role-emotional; rp: role-physical; bp: bodily pain. **b** Quality of life according to the 36-item short-form health survey questionnaire; LNF vs. LTF 10 and 20 years after operation

In comparison to a healthy control group and a standardized medically treated group (PPI) [21, 22], the score in the SF-36 questionnaire of the operated patients was elevated in all eight dimensions. In the subcategories of general health, mental health, vitality, and bodily pain, the operated cohort has even equal scores to the normalized healthy population. The SF-36 quality of life dimension scores 10 years and 20 years after operation remain constantly high. Moreover, the subjective feeling of mental health is even better (Fig. 3). The direct comparison of the quality-of-life data from the SF-36 questionnaire does not show any significant difference between the LNF group and the LTF group ten and twenty years after surgery (Fig. 3b).

**Fig. 4:**
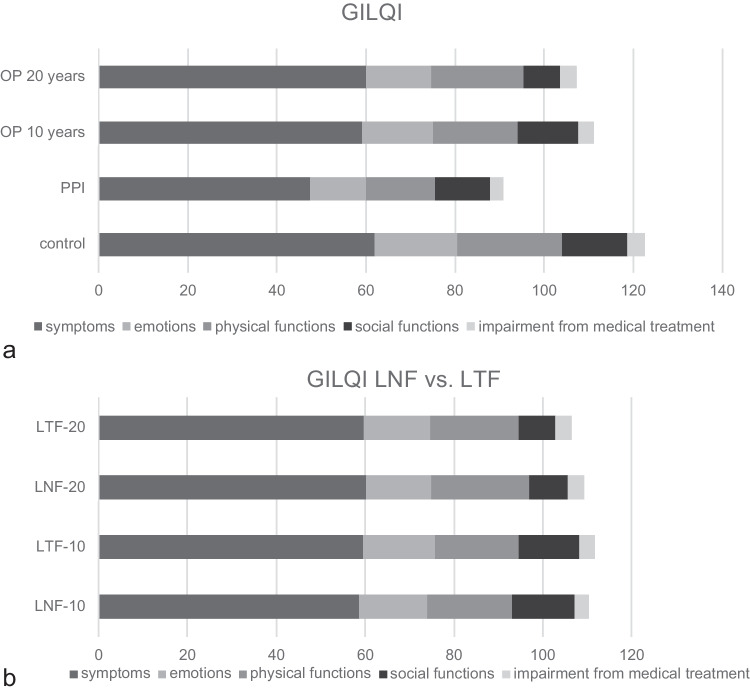
**a** Gastrointestinal Quality of Life Questionnaire (GILQI) by Eypasch et al. [17]. The maximum achievable score is 144, assembled from 5 dimensions (symptoms, physical functions, impairment from medical treatment, emotions, and social functions). The GILQI scores of operated patients (10 and 20 years after the operation) were compared to the general index of a normalized healthy population (control) and to a standardized medical treatment group (PPI) [21]. **b** Gastrointestinal Quality of Life Questionnaire (GILQI); LNF vs. LTF 10 and 20 years after operation

The mean GILQI general index of our patients ten years after anti-reflux surgery was 112,8 (+ / − 18,9), with a maximum achievable total score of 144. In the 20-year follow-up analysis, the mean GILQI general index was 107.3 (+ / − 23). The general index of a normal healthy population is 122.6 (+ / − 8,5) and 90.9 (+ / − 9,4) in a medically treated (PPI) GERD population [22] (Fig. 4a). The direct comparison of the quality-of-life data from the GILQI questionnaire shows no significant difference between the LNF group and the LTF group ten and twenty years after surgery (Fig. 4b).

#### Patient satisfaction

Ten years after surgery, 75 patients (68%) quoted the success of the operation as “excellent,” 26 patients (24%) as “good,” and four patients (4%) as “fair.” However, two patients (2%) were not pleased with the surgical result. Data is missing from three patients (3%). Twenty years after the operation, 25 patients out of the 31 who responded accepted a telephone interview. Out of them, 23 patients quoted the success of the operation as “excellent” and two patients as “good.” None of the replying patients was dissatisfied with the result.

Occasional recurrence of GERD symptoms ten years after the operation was reported in 21 patients (19%), and a redo-operation was necessary for 2 patients (2%) due to a recurrent hernia. About one-third (32%) of our cohort reported PPI treatment 10 years after surgery. The remaining two-thirds (62%) did not restart medical GERD treatment, and seven patients (6%) did not answer the question. Twenty years after the operation, three patients (12%) reported occasional recurrences of GERD symptoms; a reoperation was necessary in another two patients. 20% (five patients) are on PPI therapy, but not exclusively because of GERD.

Due to postoperative dysphagia, endoscopic balloon dilatation was done in 2 patients (2%) in the early postoperative course. Nevertheless, ten years after the operation, 97 patients (88%) would undergo a fundoplication again, if necessary. However, 20 years after surgery, sixteen (64%) patients of our cohort would choose to have a fundoplication again, whereas eight patients denied doing so, and one patient was unsure about it.

## Discussion

In this study, we focused on managing esophageal and extraesophageal GERD symptoms by anti-reflux surgery over a period of 20 years. The operation resulted in a superior quality of life compared to a historic patient cohort treated with PPI. We found out that both surgical methods (LNF and LTF) show excellent and comparable anti-reflux control in eliminating classical esophageal GERD symptoms (heartburn, regurgitation, and dysphagia). However, GERD patients receiving a Toupet fundoplication are more likely to suffer from extraesophageal GERD symptoms 10 and 20 years after surgery than patients with a Nissen fundoplication. In contrast, some patients with Nissen fundoplication report mostly mild dysphagia even 10 and 20 years after surgery.

At our center, the laparoscopic surgical method was introduced in 1994 after a two-year stay at the Creighton University of Omaha, Nebraska, with Professor Ronald Hinder. Laparoscopic Nissen fundoplication and laparoscopic Toupet fundoplication, as described above, were established in parallel, and the patients were all operated on by the same two surgeons.

In this retrospective analysis, we evaluated all patients who underwent primary laparoscopic anti-reflux surgery at our department with a follow-up time of at least 20 years. The preoperative diagnostic work-up was standardized. Symptoms, gastroscopic findings, and functional diagnosis with manometry and 24-h impedance pH monitoring were carried out regularly. Our data collection focuses on esophageal and extraesophageal symptoms and quality of life in the long term after fundoplication. However, the survey does not contain any explicit questions about the inability to belch and to vomit, gas bloating, or flatulence. Any problems with post-fundoplication symptoms would be reflected in the gastrointestinal quality-of-life survey.

Esophageal manometry six months after surgery was offered to all patients, and 117 out of 155 underwent manometry and 24-h impedance-pH monitoring. All patients showed severe GERD symptoms, with hardly any non-erosive reflux disease (NERD) patients.

In the pioneering years when the minimally invasive surgical approach was established, patients with severe GERD and a median history of symptoms of more than 10 years who were operated on. Regarding Barrett’s, there is a disproportionality in the current literature. In a review of 2019 from the Mayo Clinic, Rochester, USA, the prevalence of Barrett’s varies in the general population from 1.5 to 17% [23]. Barrett’s metaplasia in our study was defined as columnar cell metaplasia and the presence of goblet cells in histology. We attribute the high number of Barrett-positive patients to the strong selection of the most severely GERD patients in the pioneering days of minimally invasive anti-reflux surgery, thus including a high rate of Barrett’s patients. In the mid 1990s, there was no evidence on the optimal choice of surgical method, and even until today, there is ongoing discussion [24]. Based on a certain amount of experience, available data, and our own considerations at this time, it was assumed that a partial fundoplication is preferable to a total fundoplication in the case of restricted esophageal motility. Therefore, the tailored approach, i.e., the strict allocation of patients with more than 20% of defective esophageal peristalsis on manometry to the LTF group and allocation of those with normal peristalsis to the LNF group may represent a possible source of bias since motility disorder may indicate a more severe GERD.

The concept of tailoring was introduced from the beginning, and the two surgical methods were introduced in parallel at our center. All patients were operated on by the same two surgeons, so the learning curve effected the two groups in the same way.

The recording of symptoms, complaints, and feelings of patients using a self-reporting questionnaire contains methodological difficulties as qualitative data collection is converted into quantitative data. The patient’s subjective feelings depend on many factors and cannot be depicted without a doubt, whereas DeMeester score, intra-abdominal length of the esophagus, and the LESP are reproducible and measurable data. The definition of gastroesophageal reflux disease and the subdivision into esophageal and extraesophageal symptoms was made in 2006 by the Montreal consensus [25]. Differentiation and assignment of these symptoms exclusively to GERD is impossible [26]. However, eosinophilic esophagitis as a cause of dysphagia was not even established when the patients of this study were operated [27].

Back in the pioneering days of 1996, we surveyed our patients’ symptoms using a self-developed questionnaire. Being aware of the difficulty of translating the qualitative data collection, we also used standardized questionnaires to collect quality of life data (SF-36, GILQI) in addition to symptom collection. The GERD Health-Related Quality of Life (GERD-HRQL) questionnaire that is maybe best suited for this purpose was developed in 2007 and, therefore, was not applicable in our study [28].

Adherence to the follow-up protocol is always a problem when covering a period of 20 years. However, the ten-year return rate of 71% was high compared to other studies in this field. The 20-year return rate of 20%, however, was low due to some participants’ advanced age, refusal to answer, changing addresses, or deaths. Therefore, a late hernia recurrence may not be well documented. Another limitation might be the missing baseline quality of life in our cohort. However, we chose widely used standardized questionnaires allowing reference to several comparison groups of a healthy population and PPI-treated GERD patients. In our cohort, patients suffered from severe GERD with long-lasting symptoms. In most of them (96%), erosive esophagitis was diagnosed, and about 60% showed Barrett’s metaplasia.

PPI therapy after initially successful anti-reflux surgery is often reported in comparative long-term GERD studies and labeled as treatment failure [28, 29]. Still, PPI use alone is a poor marker of surgical failure. The reasons to take PPI are often elusive, and they are often prescribed for other reasons (e.g., in combination with NSAID or cortisone, gastric ulcer, dyspepsia, and bloating) as well [29]. Therefore, additive PPI treatment after operation in our cohort was not elaborated on in detail.

Long-term data on the quality of life in operated patients are rare and mostly available in individual cohort studies. A recent systematic meta-analysis (2021) and a 2015 published systematic review favor the surgical approach in GERD treatment. Anti-reflux surgery seems to be more effective than PPI treatment for GERD without significantly increasing the risk of adverse events. Long-term data (> 10 years) however are missing, and some of the trials were at high risk of bias [30, 31]. A cohort study from Sweden (2020) showed that satisfying outcomes in controlling GERD symptoms and quality of life five and ten years after surgery were maintained in a 20-year follow-up study [29]. In a 2018 published review, the perioperative mortality of a fundoplication ranged between 0.1 and 0.2%. Prolonged structural complications can occur in up to 30% of cases [32]. A recent review (2021) of surgical GERD treatment highlights a relative paucity of high-quality data, and many comparative studies are prone to bias, limiting the strength of conclusions [33]. In the Swedish study, post-fundoplication symptoms, such as bloating and flatulence, are still present in approximately 60% of patients, and 24% of patients have trouble with eructation 20 years after the operation. On the other hand, the dysphagia rate is low (3–6%) [29]. Another randomized clinical trial from Sweden (2022) comparing partial vs. total fundoplication also showed excellent GERD control with a quality-of-life improvement, at least in the physical component score and in the mental component score of the SF-36, after anti-reflux surgery over a period of 15 years. Reflux symptoms and dysphagia were recorded with the disease-specific Gastrointestinal Symptom Rating Scale (GSRS), a validated five-dimensional questionnaire [34]. Like in our study, there was no difference in quality of life between total and partial fundoplication. However, due to different reporting systems and a different focus of the study, direct comparison of the data is not valid. GSRS does not adequately reflect extraesophageal symptoms. Nevertheless, in the Swedish study, there was less dysphagia after operation and no increase over the years. The high rate of dysphagia in our cohort is probably due to the self-reporting method and definition since the patients’ quality of life is obviously not affected by this.

We found that in long-term observation, extraesophageal symptoms were not as well controlled in the LTF group compared to the LNF group. Moreover, coughing, hoarseness, and non-cardiac chest pain are clearly increasing 20 years after the operation, independent of the surgical method. Whether this is GERD-related pulmonary comorbidity, or even a physiologic consequence of aging, cannot be answered.

The exciting but unanswered question is why the quality of life improves both in the SF-36 and in the GILQI and is excellent both 10 and 20 years after surgery, although more patients report extraesophageal GERD symptoms over time. A possible explanation would be that extraesophageal GERD symptoms are rather unspecific and cannot be corelated always to GERD. Therefore, other factors, like smoking, medical side effects, chronic sinusitis, and pulmonary comorbidity, or even a physiologic consequence of aging, can also cause these symptoms. Another explanation would be that with long-term GERD, the bronchial system is pre-damaged by permanent regurgitation and acid stress, especially at the intercellular level, so that even the slightest regurgitation of gastric contents is sufficient to trigger extraesophageal symptoms. Next to this, during embryogenesis, the esophagus and the bronchial tree share the same origin, and therefore, an irritation of the esophageal mucosa can trigger pulmonary symptoms via a vagal reflex [35]. This might be more likely the case in the long-term course of the Toupet operation.

All in all, in our study, most operated-on patients were satisfied with the results 20 years after surgery, and most of them would recommend anti-reflux surgery to a relative or a friend.

Both medical and surgical guidelines recognize anti-reflux surgery as an effective treatment for GERD in appropriate patients. Unfortunately, many comparative studies on the treatment of GERD include small numbers of patients or derive from single-institutional experiences, sometimes resulting in discordant results [33].

Analyzing our data, tailoring the surgical approach based on symptoms rather than on esophageal motility could be more promising. Patients with extraesophageal symptoms may benefit from a Nissen fundoplication. For those with only classical symptoms, the thread of dysphagia in a Nissen can be omitted by performing a Toupet.

## Conclusion

The long-term quality of life > 20 years after anti-reflux surgery in GERD patients is excellent. The original idea of tailoring the operation method based on esophageal motility, out of fear of postoperative dysphagia, cannot be recommended any longer. Laparoscopic Nissen fundoplication might be superior in controlling extraesophageal reflux symptoms, but at the expense of dysphagia. In summary, tailoring the operation based on symptoms seems advantageous. Nevertheless, patients must be informed of a higher but transient dysphagia rate after Nissen fundoplication.

## Data Availability

Data relating to this study are available by contacting the corresponding author.

## Appendix

Patient self-assessment and symptom questionnaire (in German)

**Patienten—Selbsteinschätzung:**

Wie lange haben Sie schon Beschwerden?

o nie o manchmal o oft o immer Wie oft in den letzten 4 Wochen hatten Sie Schmerzen hinter dem Brustbein?

o nie o manchmal o oft o immer

Wie oft in den letzten 4 Wochen hatten Sie Sodbrennen?

o nie o manchmal o oft o immer

Wie oft in den letzten 4 Wochen kam es vor, dass Sie bereits geschluckte Speisen wieder in den Mund heraufgewürgt haben (z.B. Beim Bücken)?

o nie o manchmal o oft o immer

Wie oft in den letzten 4 Wochen hatten Sie Aufstoßen?

o nie o manchmal o oft o immer

Wie oft in den letzten 4 Wochen hatten Sie Schluckbeschwerden?

o nie o manchmal o oft o immer

Wie oft in den letzten 4 Wochen waren Sie heiser?

o nie o manchmal o oft o immer

Wie oft in den letzten 4 Wochen hatten Sie das Gefühl „einen Knödel im Hals" zu haben?

o nie o manchmal o oft o immer

Wie oft in den letzten 4 Wochen hatten Sie Asthma-ähnliche Beschwerden oder Husten, ohne erkältet zu sein?

o nie o manchmal o oft o immer

Wie oft in den letzten 4 Wochen hatten Sie Oberbauchschmerzen?

o nie o manchmal o oft o immer

Wie oft in den letzten 4 Wochen wurden Sie nachts wegen Husten, Atembeschwerden oder Sodbrennen wach?

o nie o manchmal o oft o immer

Wie oft in den letzten 4 Wochen hatten Sie ein übermäßiges Völlegefühl (Gebläht sein) nach dem Essen?

o nie o manchmal o oft o immer

Wie oft in den letzten 4 Wochen hatten Sie Durchfall?

o nie o manchmal o oft o immer

Sind Sie durch die oben genannten Beschwerden in der Ausübung Ihrer normalen Alltagstätigkeiten beeinträchtigt?

o nie o manchmal o oft o immer

Wie viele Mahlzeiten pro Tag nehmen Sie zu sich?

o 3—4 o 5—6 o > 6

Welche Speisen können Sie nicht essen und warum (Beschwerden)?

Körpergewicht:………kg. Körpergröße: …………cm

Welche Magen und Verdauungs-Medikamente nehmen Sie zurzeit ein?

Dauertherapiebei →Bedarf

0→0

0→0

0→0

0→0

Nie = nie; manchmal = ein-mehrmals/ Monat; oft = ein-mehrmals/ Woche; immer = täglich
